# Synthesis and Antitumor Activity of Novel Arylpiperazine Derivatives Containing the Saccharin Moiety

**DOI:** 10.3390/molecules22111857

**Published:** 2017-10-29

**Authors:** Hong Chen, Bing-Bing Xu, Tao Sun, Zhan Zhou, Hui-Yuan Ya, Mu Yuan

**Affiliations:** 1 College of Food and Drug, Luoyang Normal University, 6# Jiqing Road, Luoyang 471934, China; chenwexpo@sina.com (H.C.); suntao2226@163.com (T.S.); zhouzhan0406@163.com (Z.Z.); 2 Pharmaceutical Research Center, Guangzhou Medical University, 195# Dongfengxi Road, Guangzhou 511436, China; yjxubb@163.com

**Keywords:** synthesis, arylpiperazine derivatives, cytotoxic activity, CCK-8, structure-activity relationship

## Abstract

Prostate cancer is a major public health problem worldwide. For the development of potential anti-prostate cancer agents, a series of novel arylpiperazine derivatives containing the saccharin moiety based on previous studies was designed, synthesized, and evaluated in prostate (PC-3, LNCaP, and DU145) cancer cell lines for their anticancer activities. The majority of the compounds exhibited excellent selective activity for the tested cancer cells. Compounds **4** and **12** exhibited strong cytotoxic activities against DU145 cells (half maximal inhibitory concentration (IC_50_) < 2 μM). The structure–activity relationship (SAR) of these arylpiperazine derivatives was also discussed based on the obtained experimental data. This work provides a potential lead compound for anticancer agent development focusing on prostate cancer therapy.

## 1. Introduction

The selective targeting of tumor cells is the goal of modern cancer chemotherapy that is aimed at overcoming the nonspecific toxicity of most anticancer agents against normal cells [1]. At present, much of the successful cancer chemotherapy probably lies in utilizing differences in cell kinetics between tumor and normal tissue, because most drugs can show some selective toxicity toward rapidly dividing cells compared to noncycling cells [2]. Thus, drugs that are designed are expected to have high affinity to the novel targets, and they not only inhibit the proliferation but also the differentiation of tumor cells and speed up their death [3]. As the second-most common cancer worldwide for males, prostate cancer is a challenge for researchers because of the absence of any available and effective treatments. The development and progression of prostate cancer is directly related to the androgen receptor (AR) [4,5,6,7]. AR is a cytoplasmic receptor that mediates gene expression and regulates the binding of androgens, such as testosterone (T) and its active metabolite dihydrotestosterone (DHT). In order to induce their biological effects, androgens have to bind to the AR: the hormone–receptor complex binds DNA and modulates gene expression [8]. Upon androgen stimulation, the proliferation of prostate cells is increased and a malignant tumor can develop [8]. Current therapies (radical prostatectomy, chemotherapy, local radiotherapy, or hormonotherapy) are successfully applied in treating localized, androgen-dependent prostate cancer [9]. However, the treatment of hormone-refractory prostate cancer (HRPC) remains hindered by the inevitable progression of resistance to first-line treatment. Therefore, the development of novel anti-prostate cancer drugs that are effective against both the androgen-dependent and androgen-independent types of HRPC is now urgently required [10].

**Figure 1:**
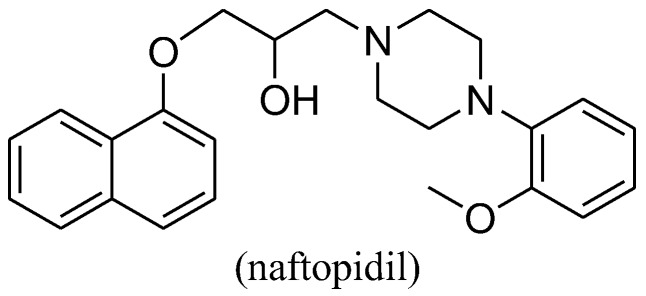
Structures of naftopidil.

Naftopidil (Figure 1), an arylpiperazine compound, is one of the most widely used α_1_-adrenergic receptor antagonists for the treatment of benign prostatic hyperplasia (BPH) [11,12]. Studies have shown that naftopidil could possibly exert an anticancer effect and inhibit prostate cancer cell growth by arresting the G1 cell cycle phase [13,14] and inducing apoptosis in malignant mesothelioma cell lines [15]. Saccharin (1,2-Benzisothiazole-3-one-1,1-dioxide) has been widely incorporated into a variety of biologically active compounds. The saccharin moiety has been identified as an important molecular component in various classes of α1a adrenergic receptor antagonists [16], 5HT1a antagonists [17], human leukocyte elastase (HLE) inhibitors [18,19,20,21,22], analgesics [23], human mast cell tryptase inhibitors [24], and aldehyde dehydrogenase inhibitors [25]. Moreover, arylpiperazine derivatives have been reported as anticancer drugs for the site-directed chemotherapy of prostate cancer in our previous works [26,27,28], and some derivatives have shown significant cytotoxic activity against the tested prostate cancer cell lines. Inspired by these, we herein report the synthesis of a series of novel arylpiperazine derivatives containing the saccharin moiety to identify new anti-prostate cancer drug candidates to treat prostate cancer. All of the synthesized compounds were evaluated for their cytotoxic activities against the androgen-insensitive human prostate cancer cell line PC-3, the androgen-sensitive human prostate cancer cell line LNCaP, the androgen-insensitive human prostate cancer cell line DU145, and the human prostate epithelial cell line WPMY-1. As we expected, compared to arylpiperazine derivatives with the 1,3-benzodioxol moiety [26] and arylpiperazine derivatives with the 3,5-dimethoxyphenoxyl moiety [27], these designed derivatives exhibited potent cytotoxic activities against the tested cancer cells (half maximal inhibitory concentration (IC_50_) < 6 μM), and displayed excellent selective activity for the tested cancer cells.

## 2. Results and Discussion

### 2.1. Chemistry

**Scheme 1:**
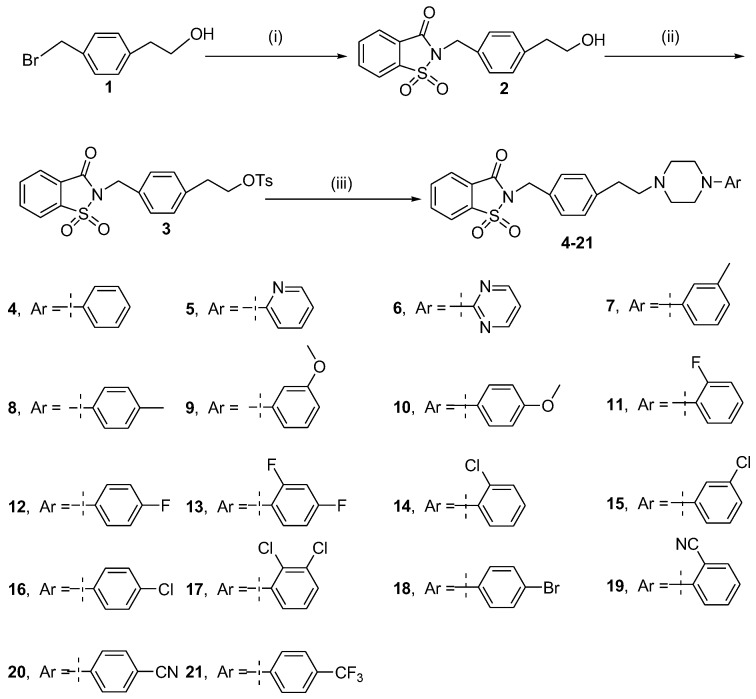
The synthesis route of derivatives **4**–**21**.

As depicted in Scheme 1, a series of novel arylpiperazine derivatives were synthesized starting from 2-(4-(bromomethyl)phenyl)ethanol **1**. First, the nucleophilic substitution reaction of compound **1** with saccharin sodium in the presence of potassium carbonate (K_2_CO_3_) gave compound **2** (85% yield) after 16 h at reflux, and then compound **2** was treated with 4-toluene-sulfonyl chloride in the presence of triethylamine and a catalytic amount of 4-dimethylaminopyridine at 0 °C for 16 h to generate compound **3** (85% yield). Finally, the reaction of compound **3** with various arylpiperazines in the presence of K_2_CO_3_ was heated at reflux for 16 h to obtain arylpiperazine derivatives **4** to **21** (Scheme 1). The structures of the compounds were confirmed using ^1^H-NMR, ^13^C-NMR, MS, and HRMS.

Reagents and conditions: (i) Saccharin Sodium, K_2_CO_3_, CH_3_CN, reflux, 16 h; (ii) TsCl, Et_3_N and 4-dimethylaminopyridine, Cl_2_CH_2_, 0 °C, 16 h; (iii) Arylpiperazines, K_2_CO_3_, CH_3_CN, reflux, 16 h.

### 2.2. Structure–Activity Relationship (SAR) Analysis for Antitumor Activity

The synthesized target compounds **4**–**21** were evaluated for their in vitro cytotoxic activities against three human prostate cancer cell lines (PC-3, LNCaP, and DU145) and compared with their effects on the human prostate epithelial cell line WPMY-1 by CCK-8 assay [29,30,31]. Naftopidil and finasteride [32] were taken as reference compounds, and the results are summarized in Table 1.

**Figure 2:**
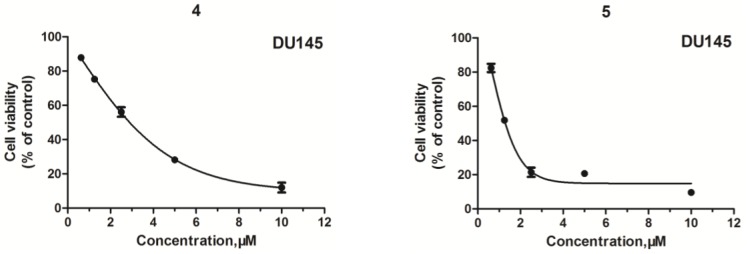

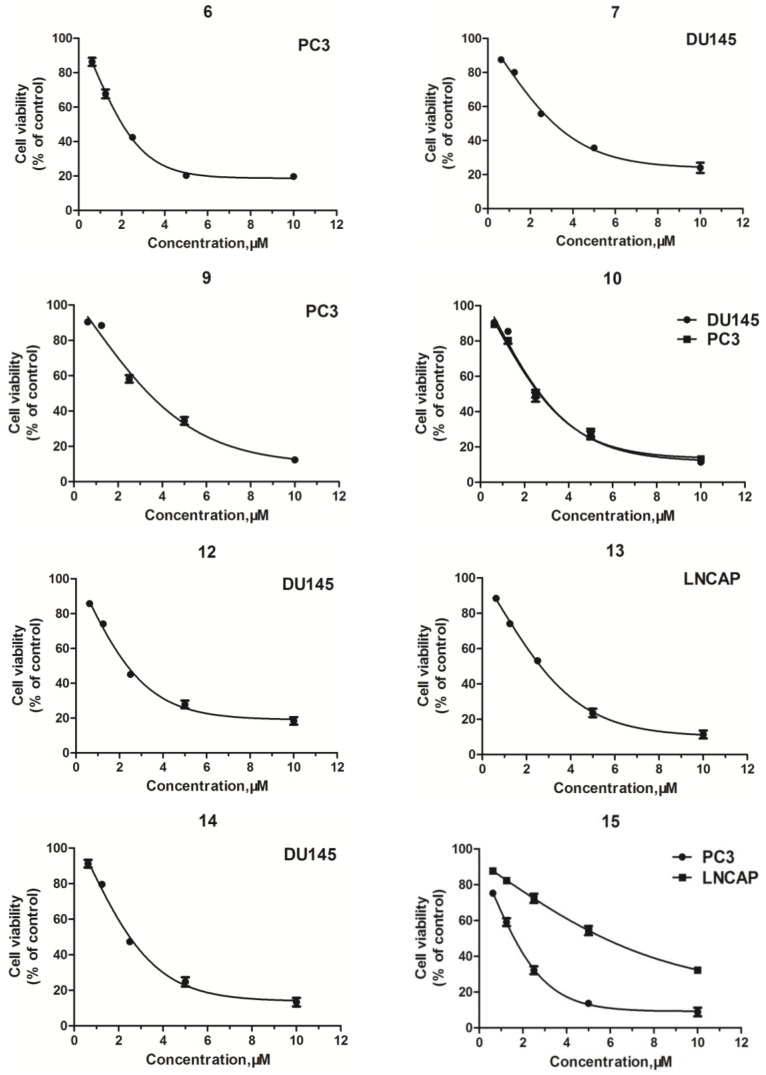

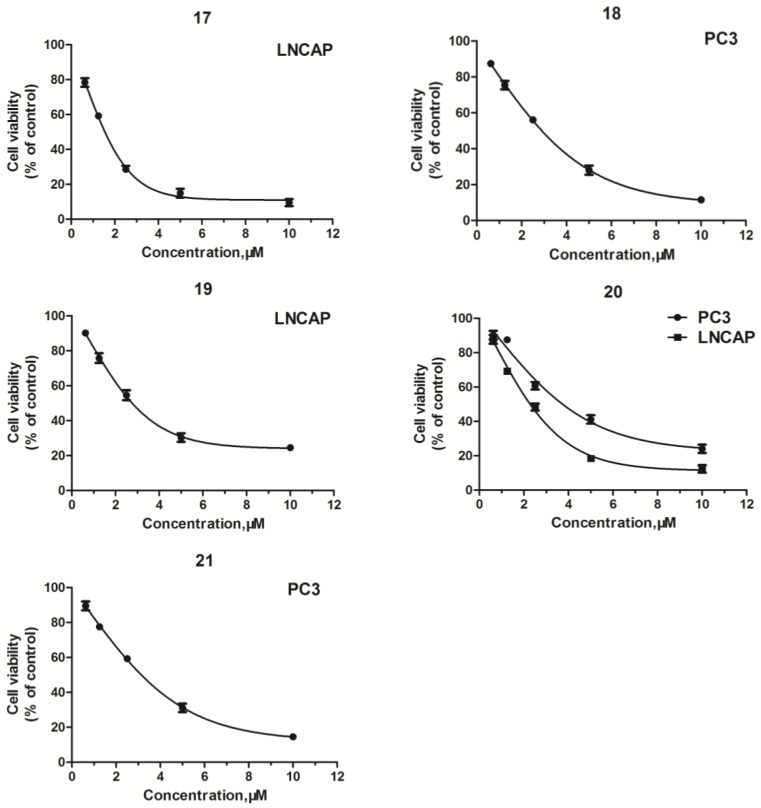
Arylpiperazine derivatives containing the saccharin moiety inhibited cell viability (percent relative to control) in the prostate cell lines PC-3, LNCaP, and DU145. All of the cells were exposed to escalating concentrations of arylpiperazine derivatives respectively for 24 h, and the cell viability was detected by CCK-8 assay.

As shown in Table 1, the majority of the compounds exhibited a more effective cytotoxic activity than the arylpiperazine derivatives with the 1,3-benzodioxol moiety [26] and the arylpiperazine derivatives with the 3,5-dimethoxyphenoxyl moiety [27] against the tested cancer cell lines, and exhibited excellent selective activity for the tested cancer cells (Figure 2). For example, the compounds **6**, **9**, and **21** exhibited excellent selective activity for PC-3 cells over other cancer cells, and the compounds **13**, **17**, and **19** exhibited excellent selective activity for LNCaP cells over other cancer cells. Moreover, the compounds **4**, **5**, **7**, **12**, and **14** exhibited excellent selective activity for DU145 cells over other cancer cells.

The SAR analysis revealed the following: (1) Compounds **4** and **5** (IC_50_ = 1.28 and 3.57 μM, respectively) exhibited strong cytotoxic activities against DU145 cells, and compound **6** (IC_50_ = 4.84 μM) displayed strong cytotoxic activities against PC-3 cells. The activity profiles indicated that the introduction of different functional groups at the 4-position of the piperazine ring was beneficial for enhancing selectivity; (2) The position of the substituent on the phenyl interestingly affected the cytotoxic activities. Amongst the compounds containing a methyl substituent, the order of the cytotoxic activities of compounds **7** (3-CH_3_) and **8** (4-CH_3_) against DU145 cells could be placed as follows: **7** > **8**. However, the compounds with electron-donating groups on the phenyl group showed another rule; for instance, the cytotoxic activities of compounds **9** (3-OCH_3_) and **10** (4-OCH_3_) against DU145 cells could be placed as follows: **10** (IC_50_ = 2.28 μM) > **9** (IC_50_ > 50 μM); (3) For PC-3 cells, compound **7** and **8** lost potency (IC_50_ > 50 μM) compared with compound **9** and **10** (IC_50_ = 5.43 and 4.38 μM, respectively). These results suggest that a methyl group on the phenyl group was inauspicious for anticancer activity; (4) Compared to compound **13** with a difluoro-substituted group, compound **12** (IC_50_ = 1.14 μM) with a fluoro group at the *p*-position on the phenyl group exhibited potent cytotoxic activities against DU145 cells and exhibited excellent selective activity for DU145 cells over other cancer cells. Moreover, compound **12** displayed weak cytotoxic effects on the human epithelial prostate normal cells WPMY-1. However, for LNCaP cells, compound **13** exhibited excellent selective activity; (5) Compounds with a chloro group at the *m*-position displayed better activity for PC-3 and LNCaP cells than did the *p*-chloro-substituted group for PC-3 and LNCaP cells, as exemplified by compound **15** (IC_50_ = 2.74 and 3.43 μM, respectively) with significantly improved activity, while compound **16** exhibited weak cytotoxic activity. Moreover, compound **17** (IC_50_ = 4.08 μM), with a dichloro-substituted group, exhibited excellent selective activity for LNCaP cells over other cancer cells, and displayed weak cytotoxic effects on the human epithelial prostate normal cells WPMY-1; (6) Compound **18** (IC_50_ = 2.25 μM) displayed better activity for PC-3 cells than the other tested compounds with the groups at the *p*-position on the phenyl group. These results indicated that a bromo group at the *p*-position on the phenyl group was beneficial for anticancer activity; (7) Compound **21** lost potency (IC_50_ > 50 μM) against LNCaP cells compared with compound **20** (IC_50_ = 3.43 μM). These results suggest that a trifluoromethyl group at the *p*-position on the phenyl group was inauspicious for anticancer activity. However, compound **21** exhibited excellent selective activity for PC-3 cells over other cancer cells.

## 3. Materials and Methods

### 3.1. Chemistry

All of the reagents and solvents used were commercially available. Solvents were dried and purified prior to use using standard procedures. Melting points were determined on SGW X-4 micro melting point apparatus (Shanghai Precision & Scientific Instrument Co., Ltd., Shanghai, China) and are uncorrected. NMR spectra were determined on a Bruker AVANCE-500 spectrometer (Billerica, MA, USA) in DMSO-*d*_6_ using TMS as an internal standard, and coupling constants (*J*) are in Hz. ESI mass spectra were recorded on an Agilent 6460 Triple Quadrupole mass spectrometer (Agilent Technologies, Santa Clara, CA, USA), and HRMS spectra were recorded on the AB Sciex 5600 Triple TOF mass spectrometer (Foster, CA, USA). Flash column chromatography was performed with silica gel (Qingdao Ocean Chemical Factory, Qingdao, China, 300–400 mesh) eluted with petroleum ether–ethyl acetate.

#### 3.1.1. Synthesis of Saccharin *N*-((2-(4-(methyl)phenyl)ethanol) (**2**)

To a solution of compound **1** (5 g, 23.30 mmol) in acetonitrile (100 mL), saccharin sodium (5.61 g, 23.30 mmol) and potassium carbonate (12.80 g, 93.20 mmol) were added, and the reaction mixture was stirred at reflux for 16 h. After cooling to ambient temperature, the reaction mixture was filtered through a Buchner funnel. After filtration, the filtrate was concentrated in vacuo and the residue was purified by silica gel column chromatography using ethyl acetate/petroleum ether (1/6, *v*/*v*) as eluent to afford 6.30 g of compound **2** as a white solid. Yield: 85%; Mp 97 °C; ^1^H-NMR (500 MHz, DMSO-*d*_6_) δ in ppm: 8.33 (d, *J* = 7.7 Hz, 1H,), 8.15–7.98 (m, 3H,), 7.33 (d, *J* = 8.0 Hz, 2H), 7.20 (d, *J* = 8.0 Hz, 2H), 4.87 (s, 2H), 3.58 (td, *J* = 7.0, 5.2 Hz, 2H,), 2.70 (t, *J* = 7.0 Hz, 2H),2.23 (t, *J* = 5.2 Hz, 1H); ^13^C-NMR (126 MHz, DMSO-*d_6_*) δ in ppm: 158.58, 139.19, 136.82, 135.87, 135.30, 132.57, 128.98, 127.78, 126.18, 125.15, 121.61, 62.01, 41.47, 38.61; MS (ESI, *m*/*z*): 318.1 [M + 1]^+^.

#### 3.1.2. Synthesis of Saccharin *N*-(4-(methyl)phenethyl 4-methylbenzenesulfonate) (**3**)

To a solution of compound **2** (5 g, 15.70 mmol), triethylamine (6.37 g, 63.10 mmol), and 4-dimethylaminopyridine (0.19 g, 1.57 mmol) in dry dichloromethane (CH_2_Cl_2_, 100 mL) at 0 °C was added dropwise a solution of 4-toluene sulfonyl chloride (4.47 g, 23.50 mmol) in CH_2_Cl_2_ (10 mL). The reaction mixture was stirred at 0 °C for 16 h. Water (30 mL) was added slowly, and the reaction mixture was extracted with CH_2_Cl_2_ (3 × 100 mL). The combined organic phase was successively washed with water and brine, dried over anhydrous magnesium sulfate, and concentrated in vacuo. The residue was purified by silica gel column chromatography using ethyl acetate/petroleum ether (1/8, *v*/*v*) as eluent to afford 5.92 g of compound **3** as a white solid. Yield: 85%; Mp 119–120 °C; ^1^H-NMR (500 MHz, DMSO-*d*_6_) δ in ppm: 8.33 (d, *J* = 7.7 Hz, 1H), 8.15–7.98 (m, 3H), 7.46 (d, *J* = 8.3 Hz, 2H,), 7.31 (d, *J* = 8.0 Hz, 2H), 7.28 (d, *J* = 8.3 Hz, 2H), 7.05 (d, *J* = 8.0 Hz, 2H), 4.51 (s, 2H), 4.16 (t, *J* = 7.0 Hz, 2H), 2.91 (t, *J* = 7.0 Hz, 2H), 2.32 (s, 3H); ^13^C-NMR (126 MHz, DMSO-*d_6_*) δ in ppm: 168.45, 144.31, 138.91, 138.25, 137.58, 132.46, 132.21, 130.42, 130.27, 129.28, 127.30, 126.83, 70.32, 48.09, 34.73, 24.32; MS (ESI, *m*/*z*): 472.1 [M + 1]^+^.

#### 3.1.3. General Procedure for the Preparation of Arylpiperazine Derivatives **4**–**21**

To a solution of **3** (100 mg, 0.23 mmol) in acetonitrile (CH_3_CN, 30 mL) was added the corresponding arylpiperazine (1.2 equiv.) and potassium carbonate (6.0 equiv.). The reaction mixture was stirred at reflux for 16 h. After cooling to ambient temperature, the reaction mixture was filtered through a Buchner funnel. After filtration, the filtrate was concentrated in vacuo and the residue was purified by silica gel column chromatography using ethyl acetate/petroleum ether (1/4, *v*/*v*) as eluent to afford the corresponding products (**4**–**21**).

Saccharin *N*-(1-(4-(methyl)phenethyl)-4-phenylpiperazine) (**4**): White solid; Yield: 70%; Mp 147–148 °C; ^1^H-NMR (500 MHz, DMSO-*d*_6_) δ in ppm: 8.33 (d, *J* = 7.7 Hz, 1H), 8.12 (d, *J* = 7.5 Hz, 1H), 8.06 (td, *J* = 7.6, 1.0 Hz, 1H), 8.01 (td, *J* = 7.6, 1.0 Hz, 1H), 7.34 (d, *J* = 8.0 Hz, 2H), 7.24 (d, *J* = 8.0 Hz, 2H), 7.19 (dd, *J* = 8.4, 7.5 Hz, 2H), 6.92 (d, *J* = 8.0 Hz, 2H), 6.76 (t, *J* = 7.6 Hz, 1H), 4.88 (s, 2H), 3.11 (t, *J* = 5.0 Hz, 4H), 2.76 (t, *J* = 7.6 Hz, 2H), 2.60–2.52 (m, 6H); ^13^C-NMR (126 MHz, DMSO-*d_6_*) δ in ppm: 159.08, 151.52, 140.57, 137.32, 136.38, 135.80, 133.09, 129.36, 129.29, 128.38, 126.69, 125.66, 122.11, 119.20, 115.79, 60.03, 53.12, 48.63, 41.96, 32.80; MS (ESI, *m*/*z*): 462.1 [M + 1]^+^; HRMS (ESI) *m*/*z* [M + 1]^+^: Calcd for C_26_H_27_N_3_O_3_S, 462.1846, found, 462.1842.

Saccharin *N*-(1-(4-(methyl)phenethyl)-4-(pyridin-2-yl)piperazine) (**5**): White solid; Yield: 45%; Mp 149–150 °C; ^1^H-NMR (500 MHz, DMSO-*d*_6_) δ in ppm: 8.33 (d, *J* = 7.7 Hz, 1H), 8.12 (d, *J* = 7.5 Hz, 1H), 8.09 (ddd, *J* = 5.0, 2.0, 1.0 Hz, 1H), 8.06 (td, *J* = 7.6, 1.0 Hz, 1H), 8.00 (td, *J* = 7.6, 1.0 Hz, 1H), 7.51 (ddd, *J* = 8.7, 7.0, 2.0 Hz, 1H), 7.33 (d, *J* = 8.0 Hz, 2H), 7.24 (d, *J* = 8.0 Hz, 2H), 6.80 (d, *J* = 8.7 Hz, 1H), 6.62 (dd, *J* = 7.0, 5.0 Hz, 1H), 4.87 (s, 2H), 3.45 (t, *J* = 5.0 Hz, 4H), 2.76 (t, *J* = 7.6 Hz, 2H), 2.56–2.50 (m, 6H); ^13^C-NMR (126 MHz, DMSO-*d_6_*) δ in ppm: 159.06, 158.58, 147.51, 140.06, 137.41, 136.82, 135.87, 135.29, 132.59, 128.78, 127.89, 126.19, 125.16, 121.61, 112.91, 107.02, 59.59, 52.43, 44.61, 41.46, 32.28; MS (ESI, *m*/*z*): 463.1 [M + 1]^+^; HRMS (ESI) *m*/*z* [M + 1]^+^: Calcd for C_25_H_26_N_4_O_3_S, 463.1798, found, 463.1794.

Saccharin *N*-(2-(4-(4-(methyl)phenethyl)piperazin-1-yl)pyrimidine) (**6**): White solid; Yield: 41%; Mp 153–154 °C; ^1^H-NMR (500 MHz, DMSO-*d*_6_) δ in ppm: 8.34 (d, *J* = 7.7 Hz, 2H), 8.32 (d, *J* = 7.5 Hz, 1H), 8.12 (d, *J* = 7.5 Hz, 1H), 8.06 (td, *J* = 7.6, 1.0 Hz, 1H), 8.01 (td, *J* = 7.6, 1.0 Hz, 1H), 7.34 (d, *J* = 8.0 Hz, 2H), 7.23 (d, *J* = 8.0 Hz, 2H), 6.61 (t, *J* = 5.0 Hz, 1H), 4.88 (s, 2H), 3.71 (br s, 4H), 2.76 (t, *J* = 7.6 Hz, 2H), 2.55–2.50 (m, 6H); ^13^C-NMR (126 MHz, DMSO-*d_6_*) δ in ppm: 161.19, 158.58, 157.86, 140.00, 136.82, 135.87, 135.29, 132.61, 128.77, 127.89, 126.19, 125.16, 121.61, 110.04, 59.51, 52.40, 43.23, 41.46, 32.19; MS (ESI, *m*/*z*): 464.1 [M + 1]^+^; HRMS (ESI) *m*/*z* [M + 1]^+^: Calcd for C_24_H_25_N_5_O_3_S, 464.1751, found, 464.1748.

Saccharin *N*-(1-(4-(methyl)phenethyl)-4-*m*-tolylpiperazine) (**7**): White solid; Yield: 20%; Mp 126–127 °C; ^1^H-NMR (500 MHz, DMSO-*d*_6_) δ in ppm: 8.33 (d, *J* = 7.7 Hz, 1H), 8.12 (d, *J* = 7.5 Hz, 1H), 8.06 (t, *J* = 7.6 Hz, 1H), 8.00 (t, *J* = 7.5 Hz, 1H), 7.34 (d, *J* = 8.0 Hz, 2H), 7.24 (d, *J* = 8.0 Hz, 2H), 7.07 (t, *J* = 7.9 Hz, 1H), 6.73 (s, 1H), 6.71 (d, *J* = 8.0 Hz, 1H), 6.58 (d, *J* = 7.5 Hz, 1H), 4.87 (s, 2H), 3.10 (t, *J* = 5.0 Hz, 4H), 2.75 (t, *J* = 7.6 Hz, 2H), 2.60–2.48 (m, 6H), 2.23 (s, 3H); ^13^C-NMR (126 MHz, DMSO-*d_6_*) δ in ppm: 159.08, 151.56, 140.58, 138.38, 137.32, 136.37, 135.79, 133.09, 129.28, 129.18, 128.38, 126.69, 125.66, 122.11, 120.04, 116.46, 113.03, 60.05, 53.15, 48.71, 41.96, 32.81, 21.89; MS (ESI, *m*/*z*): 476.1 [M + 1]^+^; HRMS (ESI) *m*/*z* [M + 1]^+^: Calcd for C_27_H_29_N_3_O_3_S, 476.2002, found, 476.2000.

Saccharin *N*-(1-(4-(methyl)phenethyl)-4-*p*-tolylpiperazine) (**8**): White solid; Yield: 43%; Mp 130–131 °C; ^1^H-NMR (500 MHz, DMSO-*d*_6_) δ in ppm: 8.33 (d, *J* = 7.7 Hz, 1H), 8.12 (d, *J* = 7.5 Hz, 1H), 8.07 (t, *J* = 7.6 Hz, 1H), 8.01 (t, *J* = 7.6 Hz, 1H), 7.35 (d, *J* = 8.0 Hz, 2H), 7.25 (d, *J* = 8.0 Hz, 2H), 7.15–7.02 (m, 1H), 6.79–6.69 (m, 2H), 6.58 (d, *J* = 7.4 Hz, 1H), 4.88 (s, 2H), 3.11 (br s, 4H), 2.79 (t, *J* = 7.6 Hz, 2H), 2.69–7.55 (m, 6H), 2.24 (s, 3H); MS (ESI, *m*/*z*): 476.1 [M + 1]^+^; HRMS (ESI) *m*/*z* [M + 1]^+^: Calcd for C_27_H_29_N_3_O_3_S, 476.2002, found, 476.2001.

Saccharin *N*-(1-(4-(methyl)phenethyl)-4-(3-methoxyphenyl)piperazine) (**9**): White solid; Yield: 50%; Mp 134–135 °C; ^1^H-NMR (500 MHz, DMSO-*d*_6_) δ in ppm: 8.33 (d, *J* = 7.7 Hz, 1H), 8.12 (d, *J* = 7.5 Hz, 1H), 8.06 (td, *J* = 7.6, 1.0 Hz, 1H), 8.01 (t, *J* = 7.6 Hz, 1H), 7.34 (d, *J* = 8.0 Hz, 2H), 7.24 (d, *J* = 8.0 Hz, 2H), 7.09 (t, *J* = 8.1 Hz, 1H), 6.51 (dd, *J* = 8.1, 2.0 Hz, 1H), 6.43 (t, *J* = 2.0 Hz, 1H), 6.35 (dd, *J* = 8.1, 2.0 Hz, 1H), 4.87 (s, 2H), 3.70 (s, 3H), 3.11 (t, *J* = 5.0 Hz, 4H), 2.75 (t, *J* = 7.6 Hz, 2H), 2.56–2.52 (m, 6H); ^13^C-NMR (126 MHz, DMSO-*d_6_*) δ in ppm: 160.64, 159.08, 152.89, 140.57, 137.32, 136.38, 135.80, 133.09, 130.03, 129.28, 128.38, 126.69, 125.66, 122.11, 108.47, 104.53, 101.87, 60.01, 55.31, 53.09, 48.60, 41.96, 32.80; MS (ESI, *m*/*z*): 492.1 [M + 1]^+^; HRMS (ESI) *m*/*z* [M + 1]^+^: Calcd for C_27_H_29_N_3_O_4_S, 492.1952, found, 492.1948.

Saccharin *N*-(1-(4-(methyl)phenethyl)-4-(4-methoxyphenyl)piperazine) (**10**): White solid; Yield: 52%; Mp 149–150 °C; ^1^H-NMR (500 MHz, DMSO-*d*_6_) δ in ppm: 8.33 (d, *J* = 7.7 Hz, 1H), 8.12 (d, *J* = 7.5 Hz, 1H), 8.06 (td, *J* = 7.6, 1.0 Hz, 1H), 8.01 (t, *J* = 7.6 Hz, 1H), 7.34 (d, *J* = 8.0 Hz, 2H), 7.23 (d, *J* = 8.0 Hz, 2H), 6.87 (d, *J* = 9.1 Hz, 2H), 6.80 (d, *J* = 9.1 Hz, 2H), 4.88 (s, 2H), 3.67 (s, 3H), 3.00 (t, *J* = 5.0 Hz, 4H), 2.75 (t, *J* = 7.6 Hz, 2H), 2.56–2.52 (m, 6H); ^13^C-NMR (126 MHz, DMSO-*d_6_*) δ in ppm: 159.08, 153.30, 145.92, 137.32, 136.38, 135.80, 133.09, 129.28, 128.38, 126.69, 125.66, 122.11, 117.74, 114.69, 60.03, 55.64, 53.22, 50.03, 41.96, 32.80; MS (ESI, *m*/*z*): 492.1 [M + 1]^+^; HRMS (ESI) *m*/*z* [M + 1]^+^: Calcd for C_27_H_29_N_3_O_4_S, 492.1952, found, 492.1949.

Saccharin *N*-(1-(4-(methyl)phenethyl)-4-(2-fluorophenyl)piperazine) (**11**): White solid; Yield: 43%; Mp 137–138 °C; ^1^H-NMR (500 MHz, DMSO-*d*_6_) δ in ppm: 8.33 (d, *J* = 7.7 Hz, 1H), 8.12 (d, *J* = 7.5 Hz, 1H), 8.06 (td, *J* = 7.6, 1.0 Hz, 1H), 8.00 (td, *J* = 7.6, 1.0 Hz, 1H), 7.34 (d, *J* = 8.0 Hz, 2H), 7.24 (d, *J* = 8.0 Hz, 2H), 7.16–7.05 (m, 2H), 7.01 (td, *J* = 8.9, 1.5 Hz, 1H), 6.98–6.90 (m, 1H), 4.88 (s, 2H), 3.00 (t, *J* = 5.0 Hz, 4H), 2.75 (t, *J* = 7.6 Hz, 2H), 2.65–2.52 (m, 6H); ^13^C-NMR (126 MHz, DMSO-*d_6_*) δ in ppm: 158.58, 155.88, 153.94, 140.08, 139.89, 139.83, 136.83, 135.87, 135.29, 132.58, 128.78, 127.88, 126.19, 125.15, 124.78, 124.75, 122.19, 122.13, 121.61, 119.14, 119.11, 115.93, 115.77, 59.49, 52.65, 50.06, 50.04, 41.47, 32.24; MS (ESI, *m*/*z*): 480.1 [M + 1]^+^; HRMS (ESI) *m*/*z* [M + 1]^+^: Calcd for C_26_H_26_FN_3_O_3_S, 480.1752, found, 480.1749.

Saccharin *N*-(1-(4-(methyl)phenethyl)-4-(4-fluorophenyl)piperazine) (**12**): White solid; Yield: 40%; Mp 152–153 °C; ^1^H-NMR (500 MHz, DMSO-*d*_6_) δ in ppm: 8.33 (d, *J* = 7.7 Hz, 1H), 8.11 (d, *J* = 7.5 Hz, 1H), 8.06 (td, *J* = 7.6, 1.0 Hz, 1H), 8.00 (td, *J* = 7.6, 1.0 Hz, 1H), 7.34 (d, *J* = 8.0 Hz, 2H), 7.23 (d, *J* = 8.0 Hz, 2H), 7.03 (t, *J* = 8.2 Hz, 2H), 6.98–6.87 (m, 2H), 4.87 (s, 2H), 3.06 (t, *J* = 5.0 Hz, 4H), 2.75 (t, *J* = 7.6 Hz, 2H), 2.57–2.52 (m, 6H); ^13^C-NMR (126 MHz, DMSO-*d_6_*) δ in ppm: 159.08, 157.35, 155.47, 148.43, 140.56, 137.32, 136.37, 135.79, 133.09, 129.28, 128.38, 126.69, 125.65, 122.11, 117.52, 117.46, 115.77, 115.59, 59.97, 53.09, 49.42, 41.96, 32.79; MS (ESI, *m*/*z*): 480.1 [M + 1]^+^; HRMS (ESI) *m*/*z* [M + 1]^+^: Calcd for C_26_H_26_FN_3_O_3_S, 480.1752, found, 480.1750.

Saccharin *N*-(1-(4-(methyl)phenethyl)-4-(2,4-difluorophenyl)piperazine) (**13**): White solid; Yield: 32%; Mp 113–114 °C; ^1^H-NMR (500 MHz, DMSO-*d*_6_) δ in ppm: ^1^H-NMR (500 MHz, DMSO) δ 8.32 (d, *J* = 7.7 Hz, 1H), 8.11 (d, *J* = 7.5 Hz, 1H), 8.06 (td, *J* = 7.6, 1.0 Hz, 1H), 8.00 (td, *J* = 7.6, 1.0 Hz, 1H), 7.35 (d, *J* = 8.0 Hz, 2H), 7.29 (d, *J* = 8.2 Hz, 1H), 7.24 (d, *J* = 8.0 Hz, 2H), 7.11 (t, *J* = 8.2 Hz, 2H), 4.88 (s, 2H), 3.01 (br s, 4H), 2.86 (t, *J* = 7.6 Hz, 2H), 2.83–7.79 (m, 6H); MS (ESI, *m*/*z*): 498.0 [M + 1]^+^; HRMS (ESI) *m*/*z* [M + 1]^+^: Calcd for C_26_H_25_F_2_N_3_O_3_S, 498.1657, found, 498.1652.

Saccharin *N*-(1-(4-(methyl)phenethyl)-4-(2-chlorophenyl)piperazine) (**14**): White solid; Yield: 40%; Mp 135–136 °C; ^1^H-NMR (500 MHz, DMSO-*d*_6_) δ in ppm: ^1^H-NMR (500 MHz, DMSO) δ 8.33 (d, *J* = 7.7 Hz, 1H), 8.12 (d, *J* = 7.6 Hz, 1H), 8.07 (t, *J* = 7.6 Hz, 1H), 8.01 (t, *J* = 7.6 Hz, 1H), 7.39 (dd, *J* = 8.0, 1.2 Hz, 1H), 7.34 (d, *J* = 8.0 Hz, 2H), 7.28 (td, *J* = 8.0, 1.2 Hz, 1H), 7.24 (d, *J* = 8.0 Hz, 2H), 7.14 (dd, *J* = 8.0, 1.2 Hz, 1H), 7.02 (td, *J* = 8.0, 1.2 Hz, 1H), 4.88 (s, 2H), 2.97 (br s, 4H), 2.75 (t, *J* = 7.6 Hz, 2H), 2.60–2.55 (m, 6H); ^13^C-NMR (126 MHz, DMSO-*d_6_*) δ in ppm: 158.58, 149.01, 140.09, 136.82, 135.87, 135.29, 132.58, 130.28, 128.78, 128.02, 127.88, 127.55, 126.19, 125.16, 123.76, 121.61, 120.80, 59.50, 52.76, 50.79, 41.47, 32.26; MS (ESI, *m*/*z*): 496.1 [M + 1]^+^; HRMS (ESI) *m*/*z* [M + 1]^+^: Calcd for C_26_H_26_ClN_3_O_3_S, 496.1456, found, 496.1454.

Saccharin *N*-(1-(4-(methyl)phenethyl)-4-(3-chlorophenyl)piperazine) (**15**): White solid; Yield: 37%; Mp 143–144 °C; ^1^H-NMR (500 MHz, DMSO-*d*_6_) δ in ppm: 8.33 (d, *J* = 7.7 Hz, 1H), 8.12 (d, *J* = 7.6 Hz, 1H), 8.06 (td, *J* = 7.6, 1.0 Hz, 1H), 8.01 (td, *J* = 7.6, 1.0 Hz, 1H), 7.34 (d, *J* = 8.0 Hz, 2H), 7.24 (d, *J* = 8.0 Hz, 2H), 7.19 (t, *J* = 8.2 Hz, 1H), 6.92 (t, *J* = 2.0 Hz, 1H), 6.88 (dd, *J* = 8.2, 2.0 Hz, 1H), 6.76 (dd, *J* = 7.9, 2.0 Hz, 1H), 4.87 (s, 2H), 3.15 (t, *J* = 5.0 Hz, 4H), 2.75 (t, *J* = 7.6 Hz, 2H), 2.56–2.52 (m, 6H); ^13^C-NMR (126 MHz, DMSO-*d_6_*) δ in ppm: 159.08, 152.75, 140.54, 137.32, 136.38, 135.80, 134.26, 133.10, 130.84, 129.29, 128.38, 126.69, 125.66, 122.11, 118.41, 114.91, 114.07, 59.95, 52.90, 48.08, 41.96, 32.77; MS (ESI, *m*/*z*): 491.1 [M + 1]^+^; HRMS (ESI) *m*/*z* [M + 1]^+^: Calcd for C_26_H_26_ClN_3_O_3_S, 496.1456, found, 496.1453.

Saccharin *N*-(1-(4-(methyl)phenethyl)-4-(4-bromophenyl)piperazine) (**16**): White solid; Yield: 42%; Mp 158–159 °C; ^1^H-NMR (500 MHz, DMSO-*d*_6_) δ in ppm: ^1^H-NMR (500 MHz, DMSO) δ 8.32 (d, *J* = 7.7 Hz, 1H), 8.11 (d, *J* = 7.5 Hz, 1H), 8.06 (td, *J* = 7.6, 1.0 Hz, 1H), 8.00 (td, *J* = 7.6, 1.0 Hz, 1H), 7.33 (d, *J* = 8.1 Hz, 2H), 7.26–7.16 (m, 4H), 6.92 (d, *J* = 9.0 Hz, 2H), 4.87 (s, 2H), 3.10 (t, *J* = 5.0 Hz, 4H), 2.74 (t, *J* = 7.6 Hz, 2H), 2.61–2.50 (m, 6H); ^13^C-NMR (126 MHz, DMSO-*d_6_*) δ in ppm: 158.58, 149.81, 140.05, 136.82, 135.87, 135.29, 132.59, 128.78, 128.54, 127.88, 126.19, 125.15, 122.19, 121.60, 116.72, 59.45, 52.44, 47.96, 41.46, 32.27; MS (ESI, *m*/*z*): 445.1 [M + 1]^+^; HRMS (ESI) *m*/*z* [M + 1]^+^: Calcd for C_26_H_26_ClN_3_O_3_S, 496.1456, found, 496.1455.

Saccharin *N*-(1-(4-(methyl)phenethyl)-4-(2,3-dichlorophenyl)piperazine) (**17**): White solid; Yield: 37%; Mp 178–179 °C; ^1^H-NMR (500 MHz, DMSO-*d*_6_) δ in ppm: ^1^H-NMR (500 MHz, DMSO) δ 8.33 (d, *J* = 7.6 Hz, 1H), 8.12 (d, *J* = 7.6 Hz, 1H), 8.07 (td, *J* = 7.6, 1.0 Hz, 1H), 8.01 (td, *J* = 7.6, 1.0 Hz, 1H), 7.34 (d, *J* = 8.0 Hz, 2H), 7.30 (d, *J* = 3.5 Hz, 2H), 7.29 (br s, 1H), 7.24 (d, *J* = 8.0 Hz, 2H), 7.14 (dd, *J* = 6.8, 3.5 Hz, 1H), 4.88 (s, 2H), 2.99 (br s, 4H), 2.76 (t, *J* = 7.6 Hz, 2H), 2.61–7.29 (m, 6H); ^13^C-NMR (126 MHz, DMSO-*d_6_*) δ in ppm: 158.59, 151.16, 140.06, 136.82, 135.88, 135.30, 132.57, 128.79, 128.41, 127.88, 126.19, 125.96, 125.16, 124.30, 121.61, 119.56, 59.43, 52.68, 50.87, 41.46, 32.23; MS (ESI, *m*/*z*): 530.0 [M + 1]^+^; HRMS (ESI) *m*/*z* [M + 1]^+^: Calcd for C_26_H_25_Cl_2_N_3_O_3_S, 530.1066, found, 530.1062.

Saccharin *N*-(1-(4-(methyl)phenethyl)-4-(4-bromophenyl)piperazine) (**18**): White solid; Yield: 42%; Mp 105–106 °C; ^1^H-NMR (500 MHz, DMSO-*d*_6_) δ in ppm: 8.33 (d, *J* = 7.7 Hz, 1H), 8.12 (d, *J* = 7.5 Hz, 1H), 8.06 (td, *J* = 7.6, 1.0 Hz, 1H), 8.00 (td, *J* = 7.6, 1.0 Hz, 1H), 7.39–7.30 (m, 4H), 7.23 (d, *J* = 8.0 Hz, 2H), 6.88 (d, *J* = 9.1 Hz, 2H), 4.87 (s, 2H), 3.11 (t, *J* = 5.0 Hz, 4H), 2.75 (t, *J* = 7.6 Hz, 2H), 2.59–2.52 (m, 6H); ^13^C-NMR (126 MHz, DMSO-*d_6_*) δ in ppm: 159.08, 150.65, 140.54, 137.31, 136.38, 135.80, 133.09, 131.90, 129.28, 128.38, 126.68, 125.66, 122.11, 117.69, 110.32, 59.95, 52.91, 48.31, 41.96, 32.77; MS (ESI, *m*/*z*): 542.0 [M + 1]^+^; HRMS (ESI) *m*/*z* [M + 1]^+^: Calcd for C_26_H_26_BrN_3_O_3_S, 540.0951, found, 540.0947.

Saccharin *N*-(2-(4-(4-(methyl)phenethyl)piperazin-1-yl)benzonitrile) (**19**): White solid; Yield: 46%; Mp 146–147 °C; ^1^H-NMR (500 MHz, DMSO-*d*_6_) δ in ppm: 8.33 (d, *J* = 7.7 Hz, 1H), 8.12 (d, *J* = 7.6 Hz, 1H), 8.07 (td, *J* = 7.6, 1.0 Hz, 1H), 8.01 (td, *J* = 7.6, 1.0 Hz, 1H), 7.69 (dd, *J* = 7.7, 1.5 Hz, 1H), 7.59 (td, *J* = 7.7, 1.5 Hz, 1H), 7.35 (d, *J* = 8.0 Hz, 2H), 7.25 (d, *J* = 8.0 Hz, 2H), 7.15 (d, *J* = 8.0 Hz, 1H), 7.08 (t, *J* = 7.6 Hz, 1H), 4.88 (s, 2H), 3.15 (t, *J* = 5.0 Hz, 4H), 2.77 (t, *J* = 7.6 Hz, 2H), 2.63–2.57 (m, 6H); ^13^C-NMR (126 MHz, DMSO-*d_6_*) δ in ppm: 158.58, 155.22, 140.03, 136.82, 135.87, 135.29, 134.29, 134.20, 132.60, 128.80, 127.89, 126.19, 125.16, 121.87, 121.61, 118.99, 118.24, 104.65, 59.34, 52.57, 51.08, 41.47, 32.21; MS (ESI, *m*/*z*): 487.1 [M + 1]^+^; HRMS (ESI) *m*/*z* [M + 1]^+^: Calcd for C_27_H_26_N_4_O_3_S, 487.1798, found, 487.1796.

Saccharin *N*-(4-(4-(4-(methyl)phenethyl)piperazin-1-yl)benzonitrile) (**20**): White solid; Yield: 48%; Mp 179–180 °C; ^1^H-NMR (500 MHz, DMSO-*d*_6_) δ in ppm: 8.33 (d, *J* = 7.7 Hz, 1H), 8.11 (d, *J* = 7.5 Hz, 1H), 8.07 (td, *J* = 7.6, 1.0 Hz, 1H), 8.00 (t, *J* = 7.6 Hz, 1H), 7.56 (d, *J* = 9.0 Hz, 2H), 7.33 (d, *J* = 8.0 Hz, 2H), 7.23 (d, *J* = 8.0 Hz, 2H), 7.01 (d, *J* = 9.0 Hz, 2H), 4.87 (s, 2H), 3.31 (t, *J* = 5.0 Hz, 4H), 2.75 (t, *J* = 7.6 Hz, 2H), 2.65–2.47 (m, 6H); ^13^C-NMR (126 MHz, DMSO-*d_6_*) δ in ppm: 158.58, 153.19, 140.00, 136.82, 135.88, 135.30, 133.26, 132.61, 128.78, 127.88, 126.18, 125.15, 121.61, 120.01, 113.99, 98.16, 59.35, 52.20, 46.31, 41.46, 32.22; MS (ESI, *m*/*z*): 487.0 [M + 1]^+^; HRMS (ESI) *m*/*z* [M + 1]^+^: Calcd for C_27_H_26_N_4_O_3_S, 487.1798, found, 487.1797.

Saccharin *N*-(1-(4-(methyl)phenethyl)-4-(4-(trifluoromethyl)phenyl)piperazine) (**21**): White solid; Yield: 46%; Mp 108–109 °C; ^1^H-NMR (500 MHz, DMSO-*d*_6_) δ in ppm: 8.33 (d, *J* = 7.7 Hz, 1H), 8.12 (d, *J* = 7.6 Hz, 1H), 8.06 (td, *J* = 7.5, 1.0 Hz, 1H), 8.01 (t, *J* = 7.5 Hz, 1H), 7.49 (d, *J* = 8.8 Hz, 2H), 7.34 (d, *J* = 8.0 Hz, 2H), 7.24 (d, *J* = 8.0 Hz, 2H), 7.05 (d, *J* = 8.8 Hz, 2H), 4.88 (s, 2H), 3.26 (t, *J* = 5.0 Hz, 4H), 2.76 (t, *J* = 7.6 Hz, 2H), 2.61–2.51 (m, 6H); ^13^C-NMR (126 MHz, DMSO-*d_6_*) δ in ppm: 159.08, 153.75, 140.52, 137.32, 136.38, 135.80, 133.11, 129.29, 128.38, 126.69, 126.61, 125.66, 122.11, 114.58, 60.22, 59.92, 52.80, 47.42, 41.96, 32.75; MS (ESI, *m*/*z*): 530.1 [M + 1]^+^; HRMS (ESI) *m*/*z* [M + 1]^+^: Calcd for C_27_H_26_N_4_O_3_S, 530.1720, found, 530.1718.

### 3.2. In Vitro Cytotoxic Assay

#### 3.2.1. Cell Culture

PC-3 and WPMY-1 cells were cultured in Dulbecco’s modified Eagle’s medium (DMEM, Invitrogen, Carlsbad, CA, USA) supplemented with 10% fetal bovine serum (FBS, Hyclone, Logan, UT, USA), 100 U/mL penicillin, and 0.1 mg/mL streptomycin (Invitrogen). DU145 cells were cultured in RPMI1640 media supplemented with 10% fetal bovine serum (FBS, Hyclone), 100 U/mL penicillin, and 0.1 mg/mL streptomycin (Invitrogen). LNCaP cells were cultured in F12 media supplemented with 10% fetal bovine serum (FBS, Hyclone), 100 U/mL penicillin, and 0.1 mg/mL streptomycin (Invitrogen). The cells were incubated at 37 °C in a humidified atmosphere with 5% CO_2_.

#### 3.2.2. Assessment of Antitumor Activity by CCK-8 Assay

Cell proliferation was measured with the Cell Counting Kit-8 (CCK-8) assay kit (Dojindo Corp., Kumamoto, Japan). Cells were harvested during their logarithmic growth phase, seeded in 96-well plates at a density of 1 × 10^5^ cells/mL, and cultured at 37 °C in a humidified incubator (5% CO_2_) for 24 h, followed by exposure to various concentrations of compounds tested for 24 h. Subsequently, 10 μL of CCK-8 (Dojindo) was added to each well, and the cells were then incubated for an additional 1 h at 37 °C to convert WST-8 into formazan. Cell growth inhibition was determined by measuring the absorbance (Abs) at λ = 450 nm using a microplate reader. Three independent experiments were performed. Cell growth inhibition was calculated according to the following equation:

Growth inhibition = (1 − OD of treated cells / OD of control cells) × 100%

OD = optical density

The half maximal inhibitory concentrations (IC_50_) were obtained from a linear regression analysis of the concentration-response curves plotted for each tested compound.

## 4. Conclusions

In conclusion, this paper has reported the synthesis and biological evaluation against three human prostate cancer cells and human prostate epithelial cells of a novel class of arylpiperazine derivatives containing the saccharin moiety. The majority of the compounds exhibited excellent selective activity for the tested cancer cells. Compounds **4** and **12** exhibited strong cytotoxic activities against DU145 cells (IC_50_ < 2 μM). The SAR analysis revealed that compounds with a group at the *p*-position on the phenyl group exhibited potent cytotoxic activities against the tested cancer cells. Results from this study could serve as a valuable guideline for further research on novel arylpiperazine derivatives. Further research involving another novel class of arylpiperazine derivatives is in progress.

## Figures and Tables

**Table 1:** In vitro cytotoxicity of compounds **4**–**21**.

Compd.	IC_50_ (μM) ^a^			
	PC-3 ^b^	LNCaP ^b^	DU145 ^b^	WPMY-1 ^b^
**4**	>50	>50	1.28 ± 0.04	>50
**5**	>50	>50	3.57 ± 0.08	>50
**6**	4.84 ± 0.17	>50	>50	48.27 ± 0.56
**7**	>50	>50	3.65 ± 0.10	>50
**8**	>50	>50	>50	ND ^c^
**9**	5.43 ± 0.18	>50	>50	>50
**10**	4.38 ± 0.13	>50	2.28 ± 0.05	>50
**11**	>50	47.46 ± 2.17	>50	>50
**12**	>50	>50	1.14 ± 0.10	>50
**13**	>50	5.03 ± 0.13	>50	>50
**14**	14.57 ± 1.12	>50	3.39 ± 0.11	ND ^c^
**15**	2.74 ± 0.11	3.43 ± 0.16	>50	>50
**16**	>50	>50	>50	ND ^c^
**17**	>50	4.08 ± 0.15	>50	>50
**18**	2.25 ± 0.07	>50	9.05 ± 0.23	>50
**19**	>50	5.14 ± 0.16	>50	39.15 ± 0.17
**20**	2.66 ± 0.04	3.43 ± 0.10	>50	46.34 ± 0.51
**21**	3.73 ± 0.08	>50	>50	ND ^c^
Naftopidil	42.10 ± 0.79	22.36 ± 0.61	34.58 ± 0.31	>50
Finasteride	17.83	14.53	13.53	−

^a^ Half maximal inhibitory concentration (IC_50_) values are taken as means ± standard deviation from three experiments; ^b^ PC-3, LNCaP, and DU145, human prostate cancer cell lines; WPMY-1, the human prostate epithelial cell line; ^c^ ND = not determined.
